# Glucose-6-phosphate dehydrogenase deficiency, chlorproguanil-dapsone with artesunate and post-treatment haemolysis in African children treated for uncomplicated malaria

**DOI:** 10.1186/1475-2875-11-139

**Published:** 2012-07-10

**Authors:** Carine Van Malderen, Jean-Pierre Van Geertruyden, Sonia Machevo, Raquel González, Quique Bassat, Ambrose Talisuna, Adoke Yeka, Carolyn Nabasumba, Patrice Piola, Atwine Daniel, Eleanor Turyakira, Pascale Forret, Chantal Van Overmeir, Harry Van Loen, Annie Robert, Umberto D’ Alessandro

**Affiliations:** 1 Faculté de pharmacie et des sciences biomédicales, Université catholique de Louvain, Brussels, Belgium; 2 International Health Unit, University of Antwerp, Antwerp, Belgium; 3 Centro de Investigação em Saúde da Manhiça (CISM), Maputo, Mozambique; 4 Barcelona Centre for International Health Research (CRESIB), Hospital Clínic-Universitat de Barcelona, Barcelona, Spain; 5 Department of Epidemiology and Biostatistics, Makerere University School of Public Health, P.O Box 7072, Kampala, Uganda; 6 Malaria Public Health and Epidemiology Group (MPHEG), University of Oxford-KEMRI-Wellcome Trust Research Program, Nairobi, Kenya; 7 Epicentre Mbarara Research Base, Mbarara, Uganda; 8 Institute of Tropical Medicine, Antwerp, Belgium; 9 Université catholique de Louvain. Brussels Health Sector – Institut de recherche expérimentale et clinique Pôle, Epidémiologie et biostatistique B1.30.13, Brussels, Belgium

**Keywords:** Malaria, Artemisinin-based combination therapy, Chlorproguanil-dapsone, Artesunate, Glucose-6-phosphate dehydrogenase deficiency, Uganda, Mozambique, Restriction fragment length polymorphisms, Conditional logistic regression

## Abstract

**Background:**

Malaria is a leading cause of mortality, particularly in sub-Saharan African children. Prompt and efficacious treatment is important as patients may progress within a few hours to severe and possibly fatal disease. Chlorproguanil-dapsone-artesunate (CDA) was a promising artemisinin-based combination therapy (ACT), but its development was prematurely stopped because of safety concerns secondary to its associated risk of haemolytic anaemia in glucose-6-phosphate dehydrogenase (G6PD)-deficient individuals. The objective of the study was to assess whether CDA treatment and G6PD deficiency are risk factors for a post-treatment haemoglobin drop in African children <5 years of age with uncomplicated malaria.

**Methods:**

This case–control study was performed in the context of a larger multicentre randomized clinical trial comparing safety and efficacy of four different ACT in children with uncomplicated malaria. Children, who after treatment experienced a haemoglobin drop ≥2 g/dl (cases) within the first four days (days 0, 1, 2, and 3), were compared with those without an Hb drop (controls). Cases and controls were matched for study site, sex, age and baseline haemoglobin measurements. Data were analysed using a conditional logistic regression model.

**Results:**

G6PD deficiency prevalence, homo- or hemizygous, was 8.5% (10/117) in cases and 6.8% (16/234) in controls (p = 0.56). The risk of a Hb drop ≥2 g/dl was not associated with either G6PD deficiency (adjusted odds ratio (AOR): 0.81; p = 0.76) or CDA treatment (AOR: 1.28; p = 0.37) alone. However, patients having both risk factors tended to have higher odds (AOR: 11.13; p = 0.25) of experiencing a Hb drop ≥2 g/dl within the first four days after treatment, however this finding was not statistically significant, mainly because G6PD deficient patients treated with CDA were very few. In non-G6PD deficient individuals, the proportion of cases was similar between treatment groups while in G6PD-deficient individuals, haemolytic anaemia occurred more frequently in children treated with CDA (56%) than in those treated with other ACT (29%), though the difference was not significant (p = 0.49).

**Conclusion:**

The use of CDA for treating uncomplicated malaria may increase the risk of haemolytic anaemia in G6PD-deficient children.

## Background

Chlorproguanil–dapsone (CD) is a fixed-dose anti-folate combination that was developed by a public-private partnership for the treatment of *Plasmodium falciparum* uncomplicated malaria [1]. CD received approval from the UK Regulatory Agency in July 2003 for the treatment of uncomplicated *falciparum* malaria in non-pregnant adults. Data from the phase III clinical programme conducted in children in sub-Saharan Africa showed that CD achieved significantly higher cure rates compared to sulphadoxine-pyrimethamine (SP) and was well tolerated [2]. Importantly, CD was active against SP-resistant parasites in Africa [3,4]. However, and following the significant reduction of haemoglobin (Hb) observed in patients with glucose-6-phosphate dehydrogenase (G6PD) deficiency after treatment with CD and artesunate (CDA) in two phase III trials [1,5], clinical research on this product was stopped and all CD doses already on the market withdrawn [6].

Dapsone is metabolized in a hydroxylamine metabolite that is susceptible to trigger oxidation damages in red blood cells, leading, if not thwarted by a protection mechanism, to haemolytic anaemia [7]. This is a concern in G6PD-deficient individuals. This genetic polymorphism is particularly prevalent in malaria-endemic areas, and affects about 400 million people worldwide [8].

G6PD, a cytoplasmic enzyme expressed in all cells, catalyses the first step of the pentose phosphate pathway and plays an essential role in protecting red blood cells from oxidative stress [9]. The G6PD gene is located on the chromosome X, meaning that it mainly affects males. The G6PD gene is highly polymorphic; more than 400 allelic variants have been described, including 34 different mutations with an allelic frequency higher than 1% [10,11]. According to biochemical characteristics and to genome mutations, G6PD gene is considered as tri-allelic in sub-Saharan Africa: (1) G6PD type B, the most common allelic form, coupled with a normal enzymatic activity, (2) G6PD type A with one point mutation A376G (Asn → Asp) and 85% activity and (3) G6PD type A- with two point mutations A376G and G202A (Val → Met) and 12% activity [12]. In some cases, the second mutation is either G680T or T968G. Other mutations have been occasionally reported, such as A- Betica or A- Sierra Leone [13]. In tropical Africa, G6PD type A- (class III) represents 90% of all G6PD deficiencies.

In a G6PD-deficient individual, oxidative stress can seriously damage red blood cells (reactive oxygen species generation, partial Hb denaturation, Heinz bodies production, membrane function alteration), resulting in acute haemolysis. When acute haemolysis is triggered by a treatment such as CD, Hb drop can be of about 2 g/dl within one or two days after treatment administration [14]. Unlike G6PD Mediterranean, a more severe deficiency in which haemolysis continues until well after the administration of drug is stopped, the haemolytic anaemia caused by G6PD A- is self-limited because only the older red blood cells are destroyed [9]. Epidemiological and *in vitro* studies suggested that G6PD deficiency could confer a protection against *P. falciparum* infection by inhibiting erythrocyte invasion or intracellular development of the malaria parasite [12]. Given the current recommendation to use artemisinin-based combination therapy (ACT), in which an artemisinin derivative is combined with another anti-malarial drug, CDA was developed as a low-cost, simple, fixed-dose ACT for use in Africa [1]. However, results of a recent multicentre trial comparing CDA to CD alone showed that, despite its better efficacy, CDA haemolytic risk in G6PD-deficient patients did not allow further use of this treatment [1]. Nevertheless, a multicentre trial including non-co-formulated CDA as a study treatment had already started and a few hundred children had already been included before the decision to stop CDA development was taken. The objective of the study was to assess whether CDA treatment and G6PD deficiency constitute risk factors for a post-treatment Hb drop in African children <5 years of age with uncomplicated malaria.

## Methods

### Study site and population

This study was part of a large multicentre trial (seven African countries, 12 sites) testing the safety and efficacy of four ACT for uncomplicated malaria in children, namely dihydroartemisinin-piperaquine (DHAPQ), amodiaquine-artesunate (AQ + AS), artemether-lumefantrine (AL), and CDA (Trial Registration NCT00393679 [15]). Details of the study methods are described elsewhere [16].

Briefly, CDA was compared with DHAPQ and AL in Rwanda (Rukara, Mashesha) and in Uganda (Jinja, Tororo) and with DHAPQ and AQ + AS in Uganda (Mbarara) and Mozambique (Manhiça). Rwandan samples were not included in the series as they were not available at the time of the analysis. Children aged 12–59 months old with uncomplicated malaria were randomly allocated to one of the three study arms if they fulfilled the following inclusion criteria: body weight >5 kg; *P. falciparum* mono-infection with density between 2,000 and 200,000/μl; fever (axillary temperature ≥37.5°C) or history of fever in the last 24 hours, and Hb ≥7 g/dl. Exclusion criteria were: participation within the last month in any other investigational drug study; known hypersensitivity to at least one of the study drugs; severe malaria [17] or other danger signs; severe malnutrition or any other illness or condition (cardiac, renal, hepatic diseases) that would interfere with the study results, including known G6PD deficiency; contra-indication to receive the trial drugs; or ongoing prophylaxis with drugs having anti-malarial activity. Eligible patients were allocated one of the four study drugs according to a randomization list stratified by country, with each treatment allocation concealed in opaque sealed envelopes that were opened only after patient’s enrolment.

### Intervention

Treatment was administered under direct supervision during three consecutive days and according to the patient’s body weight. CD was orally administered at a dose of 2.0 mg/kg of chlorproguanil and 2.5 mg/kg of dapsone once a day during three days, using commercial Lapdap® paediatric tablets containing each 15 mg of chlorproguanil and 18.75 mg of dapsone. Arsumax® tablets (Guilin Pharmaceutical Co. Ltd. Guangxi for Sanofi-Synthelabo, Gentilly, France) containing 50 mg of artesunate were given at the dosage of 4 mg/kg/day during three days. The other formulations of ACT were administered according to the manufacturers’ instructions [16].

### Follow-up

All children were kept at the health facility during the three-day dosing period. The mother/guardian was asked to return with the child for scheduled visits on days 3, 7, 14, 21 and 28 post-treatment, or if any symptoms occurred between scheduled visits. Capillary or venous blood was taken at each visit. The follow-up description can be found in the main study methods [16].

### Parasite density and hb measurements

Thick and thin blood films were prepared, dried and Giemsa stained; parasite density was estimated by counting the number of asexual parasites in 200 white blood cells (WBC), assuming a standard WBC count of 8,000/μl. Hb was measured using HemoCue® device at day 0, before treatment, and using an haematology analyser (ABX Pentra 60 in Mbarara & Sysmex KX-21N in Manhiça, Jinja and Tororo) at days 1, 2, 3, 7, and 28. Three blood spots were collected on filter paper (Whatman Grade 3) at enrolment and at each visit from day 7 onwards. Each filter paper was dried and individually stored in a plastic bag containing silica gel. All filter papers were subsequently transferred to the Institute of Tropical Medicine (Antwerp, Belgium) for genotyping.

### G6PD type A- detection

DNA was extracted from dried blood spot on filter paper with the Chelex method [18]. Considering that type A- is the most common allele in sub-Saharan Africa, the presence of the G202A mutation was assessed by polymerase chain reaction (PCR)-restriction fragment length polymorphisms (RFLP) [19,20]. Sequences containing mutation site were amplified by PCR using following primers: CAG-CCA-CTT-CTA-ACC-ACA-CAC-CT (forward) and CCG-AAG-TTG-GCC-ATG-CTG-GG (reverse) [20]. PCR conditions were: MgCl_2_ 1.5 mM, dNTP 0.6 mM, each primer 0.2 μM, Taq polymerase 0.02U/μl, DNA 1 μl, buffer 1x and Milli-Q water. Cycler programme steps were: primary denaturation 5’ at 95°C, denaturation 1’ at 95°C, annealing 45” at 64°C, extension 1’ at 72°C, 34 cycles, and final extension 5’ at 72°C. The 202 mutation was detected with the NlaIII restriction enzyme that cleaves DNA only if mutation is present. RFLP conditions were: NlaIII 0.08U/μl, BSA 1x, buffer, Milli-Q water and 5 μl of the PCR product. After eight hours of incubation at 37°C, enzyme was inactivated 20’ at 65°C. Digested DNA samples were run in a 2% agarose gel by electrophoresis in Tris-Acetate-EDTA (TAE), marked with ethidium bromide and revealed with UV. Results were interpreted as followed: one band (338 bp) corresponds to B or A genotype, two bands (215 bp, 123 bp) correspond to A- genotype, and three bands (338 bp, 215 bp, 123 bp) correspond to heterozygotes (BA- or AA- genotype).

### Data analysis

A matched case control approach was used. Children with a Hb drop ≥2 g/dl within three days post-treatment were considered as cases and all others as controls. As the number of participants with a Hb drop was low, two matched controls were selected for each case in order to increase the power of the analysis. Controls were matched for study site, sex, age (<10 months difference) and Hb at baseline (<1 g/dl difference). Proportions were compared using chi-square or Fisher exact tests and continuous variables were compared using Student’s *t* test. The geometric mean of the parasite density was used as threshold between “high” and “low” parasitaemia. Mild anaemia was defined as Hb <11 g/dl. Data were analysed using NCSS software (Number Cruncher Statistical Systems 2004/PASS 2005, Kaysville, UT, USA). A conditional logistic regression model was built using STATA version 9 (Stata Corporation, College Station, TX). Multi-colinearity was checked and no variance inflation factor was greater than 10. Stepwise multivariate analyses were performed, and all possible interactions up to order 2 were tested, checking at each step how effects and likelihood were modified (likelihood ratio test and Akaiké information criterion, AIC). Adjusted odds ratios (AORs) and standard errors (SE) were calculated taking into account the interaction term. A p-value <0.05 was considered as significant; all p-values are 2-sided and reported confidence intervals (CI) are 95%CI.

## Results

### Post-treatment haemolysis

Hb data were not available for at least one of the first four days for 127 patients and 107 Rwandan samples were not available. Among the remaining 571 patients, Hb dropped by ≥2 g/dl in 117 (20.5%). Mean Hb decrease, compared to day 0, was 0.57 g/dl (CI: 0.47-0.60) at day 1, 0.76 g/dl (CI: 0.67-0.85) at day 2 and 0.51 g/dl (CI: 0.43-0.59) at day 3. In most cases (43%, 50/117), the ≥2 g/dl drop occurred at day 2. Haematological recovery was observed commencing at day 3 onwards, with a mean Hb of 11 g/dl (CI:10.9-11.1) at day 28. In one child Hb dropped from 6.5 g/dl at day 0 to 3.3 g/dl at day 7, requiring a blood transfusion. The child was G6PD heterozygote and treated with CDA.

The lowest percentage of patients with a Hb ≥2 g/dl drop was observed in Tororo in Uganda (Table 1, p = 0.015), which also showed the lowest mean age (26.6 months *vs* 33.8, 32.7 and 31.1 in Manhiça, Mbarara and Jinja, respectively, p = 0.0001) The Hb ≥2 g/dl drop was more frequent in children under 28 months (p = 0.026) with a high parasite density (p = 0.034), and without mild anaemia (p < 0.001). Percentage of patients whose Hb dropped by ≥2 g/dl did not differ between treatment groups (Table 1).

**Table 1:** Percentage of patients whose Hb dropped by ≥2 g/dl (cases), by study site and risk factors

	**ΔHb ≥ 2 g/dl (%)**		p-value*
**Site**			
Mozambique Manhiça	23.4	(49/209)	0.015
Uganda Mbarara	23.8	(41/172)	
Uganda Tororo	8.6	(8/93)	
Uganda Jinja	19.6	(19/97)	
**Sex**			
Boys	21.1	(64/303)	0.691
Girls	19.8	(53/268)	
**Age, months**			
12–27	35.3	(38/248)	0.026
28–43	25.1	(51/203)	
44–60	23.3	(28/120)	
**Mild anaemia (Hb < 11 g/dl)**			
Yes	13.9	(63/452)	<0.001
No	45.4	(54/119)	
**Parasite density**			
Low (<29623 μl^-1^)	15.5	(39/251)	0.034
High (>29623 μl^-1^)	24.4	(78/320)	
**Treatment**			
CDA	22.0	(42/191)	0.312
AL	13.1	(8/61)	
DHAPQ	18.9	(36/190)	
AQ + AS	24.0	(31/129)	

CDA = chlorproguanil-daspone + artesunate. AL = artemether-lumefantrine. DHAPQ = dihydroartemisinin-piperaquine. AQ + AS = amodiaquine-artesunate.

*Chi-square test.

### G6PD deficiency

**Figure 1:**
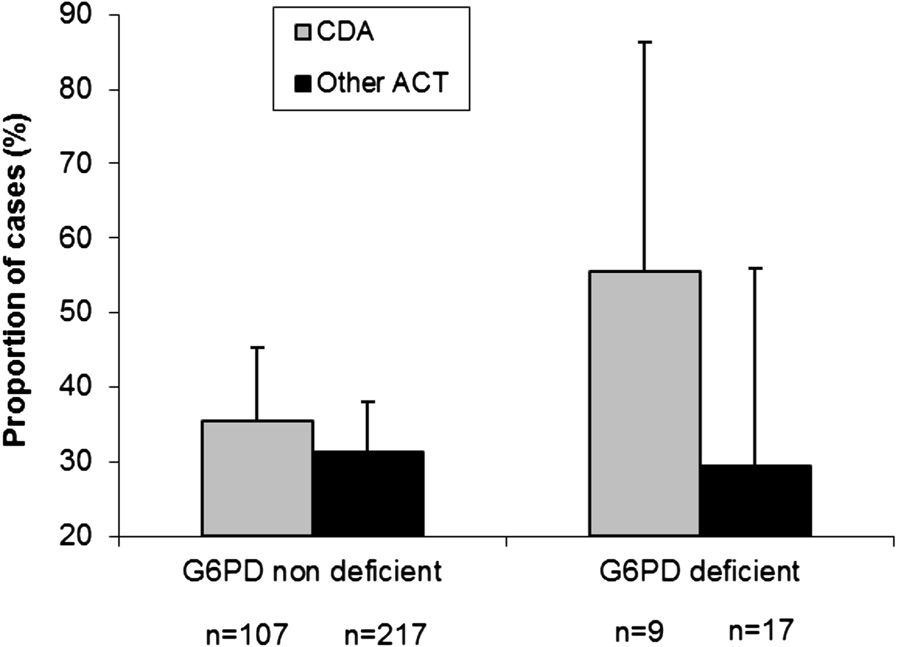
**Proportion of children with a Hb drop ≥2 g/dl (cases) across G6PD status and treatment groups.** CDA = chlorproguanil-dapsone + artesunate. Other ACT = artemether-lumefantrine, dihydroartemisinin-piperaquine, amodiaquine-artesunate.

Patients’ recruitment started in the different sites between July and October 2007 and CDA allocation was stopped in February 2008. At the time the CDA arm was stopped, 805 patients had already been included in the sites evaluating CDA. One hundred and seventeen cases could be matched to 234 controls using a ratio 1:2 (Table 2). G6PD deficiency (homozygous A-) was found in 8.5% (10/117) of cases and in 6.8% (16/234) of controls (p = 0.56). As expected, homozygous girls represented only a small proportion (7.7%, 2/26) of deficient subjects and were absent from the control group. Nearly one in four girls carried the A- mutation as heterozygous (24%, 40/165). In boys, 13% (24/186) were hemizygous, with no difference between cases and controls. The proportion of deficient A- subjects varied across the study sites: 3.3% (CI: 0.9-8.3) in Mbarara, 8.3%; (CI: 1.0-27.0) in Tororo, 14.8% (CI: 6.6-27.1) in Jinja, Uganda and 7.8% (CI: 4.1-13.3) in Manhiça, Mozambique. In non-G6PD deficient individuals, the proportion of cases was similar between treatment groups (AQ + AS: 35%, AL: 26%, CDA: 35%, DHAPQ: 31%, p = 0.75). However, in G6PD-deficient individuals, haemolytic anaemia occurred more frequently in children treated with CDA (5/9) than in those treated with other ACT though the difference was not significant (Figure 1, p = 0.49). The five cases in the 17 deficient children treated with another ACT were distributed as follows: 2/4 in AQ + AS, 1/1 in AL and 2/12 in DHAPQ.

**Table 2:** Comparison of G6PD status between cases and controls matched for study site, sex, age and Hb at baseline (%)

	**Cases** **ΔHb ≥ 2 g/dl**		**Controls** **ΔHb < 2 g/dl**	
	**(n = 117)**		**(n = 234)**	
Deficient A-*				
Homozygous girls	2	(1.7)	0	(0.0)
Hemizygous boys	8	(6.8)	16	(6.8)
**Heterozygote A-**				
Girls	10	(8.5)	30	(12.8)
**Normal**				
Boys	54	(46.1)	108	(46.1)
Girls	43	(36.7)	80	(34.2)

* defined as a detection of mutation 202 in amplified DNA.

### Effects of G6PD deficiency and CDA on post-treatment haemolysis risk

In assessing the effect of G6PD deficiency and CDA treatment on a Hb ≥2 g/dl drop, all plausible interactions were considered but only the interaction CDA*G6PD deficiency was retained because, even if non-significant, effects were modified when the interaction was deleted. This indicates that, in terms of Hb decrease, CDA treatment effect was different between G6PD-deficient and normal children. The final conditional logistic regression model (Table 3) included Hb at baseline, log of parasite density, G6PD deficiency, CDA and the CDA*G6PD deficiency interaction, as multivariate risk factors for Hb drop by ≥2 g/dl. G6PD deficient individuals treated with CDA had higher odds of experiencing a Hb drop of ≥2 g/dl than non-G6PD deficient individuals treated with another ACT (OR = 11.13, CI: 0.63-197), though it did not reach statistical significance (Table 3).

**Table 3:** Uni- and multivariate determinants of haemolytic anaemia in 12–59 months children (n = 351) treated for a clinical malaria, using conditional logistic regression

**Variable**	**OR (95%CI)**	**p-value**	**AOR (95%CI)**	**p-value**
Baseline Hb in g/dl	7.27 (3.63–14.54)	<0.001	7.05 (3.6–13.88)	<0.001
Log parasite density/μl	1.29 (1.06–1.59)	0.012	1.17 (0.93–1.47)	0.17
A- G6PD deficiency (vs. G6PD normal)	1.32 (0.54–3.23)	0.54	0.81^§^ (0.21–3.08)	0.76
CDA (vs. other ACT^†^)	1.25 (0.79–1.99)	0.34	1.28^§^ (0.74–2.22)	0.37
A- G6PD and CDA (vs. G6PD normal and other ACT)*			11.13 (0.63–197)	0.25

OR = Odds Ratio. AOR = Adjusted Odds Ratio. CI = Confidence Interval.

^†^ artemether-lumefantrine, dihydroartemisinin-piperaquine, or amodiaquine-artesunate.

^§^ AOR value when the other co-variable in the interaction term (CDA or G6PD) equals 0.

* AOR, 95% CI and p-value were computed using the Wald χ² statistic.

## Discussion

Neither CDA treatment nor G6PD alone were risk factors for post-treatment haemolysis though children with both factors seemed to be more likely to experience, within the first four days after starting the treatment, a Hb drop of ≥2 g/dl. These results are consistent with other reports on CD. In a multicentre randomized trial that included 1,850 children with uncomplicated *falciparum* malaria, G6PD-deficient malaria patients treated with CD had a Hb drop at day 7 that was not observed in G6PD-normal patients [2]. In Rwanda [21], G6PD-deficient subjects treated with CDA had a significantly higher risk for haemolytic anaemia than those treated with amodiaquine and artesunate [22]. In the phase III trial comparing CDA with artemether-lumefantrine [6,5], both CDA and G6PD deficiency were independently associated with the occurrence of a Hb composite safety endpoint comprising the occurrence of a Hb decrease of ≥ 40 g/L or ≥40% *vs* baseline or Hb <50 g/L or blood transfusion. Occurrence of this Hb composite safety endpoint in G6PD deficient patients was 35% with CDA and 0% with AL. G6PD deficiency was also reported by Tiono *et al.*[1] as the main significant risk factor for a Hb safety endpoint in patients treated with CDA or CD alone. Though using different methods and studying different age populations, all aforementioned studies illustrated that the impact of dapsone (CDA or CD) on Hb level occurred mainly in G6PD deficient patients (i.e. the effect of the interaction between the CD(A) treatment and G6PD deficiency).

Observations on heterozygotes are difficult to interpret because they have two red blood cells populations and their proportion varies between individuals [14]. Heterozygotes were considered as G6PD-normal patients on the assumption that type A-, a moderate G6PD deficiency (of class III), could barely trigger clinical manifestations if only a part of red blood cells was affected. Nevertheless, heterozygotes having large proportion of deficient red blood cells may have been misclassified as normal.

The biomolecular approach used is appropriate when G6PD-deficiency type is known. In sub-Saharan Africa, G6PD type A- is extremely common but can be caused by several mutations. Genotypes A- with different mutations than that tested in this study (G202A) have been reported. It is estimated that the A376G-G680T and A376G-T968G genotypes represent less than 5% of type A- deficiencies, though in Senegal they are more frequent than the usual A376G-G202A [23]. There are no data on the genotypes A- related mutations in Mozambique and Uganda so that some patients in these sites may have been misclassified in terms of G6PD status. Matching allowed controlling for site, sex and age in the analysis. However, as Hb at baseline was not completely matched, it was kept in the model as an independent predictor. Haemoglobin drop ≥2 g/dl criterion in the first days after treatment was determined according to Beutler’s observations [9]. In children, the lowest haemoglobin value is usually observed at day 3 after treatment [2,24]. The composite Hb safety enpoint would not have been applicable in the present study as only 10 children would have met this criterion (taking observations from day 0 to day 7). In this sample, mean Hb decrease from baseline did not exceed 1 g/dl at any day. Therefore the ≥2 g/dl criterion was judged sufficient to select the “severe” post-treatment haemolysis. Body temperature was excluded from the model because it was judged less reliable. The choice of the reported analyses was based on the assumption that in G6PD deficient children, the proportion of cases would be higher in children treated with CDA than in children treated with other ACT. Sample sizes of nine deficient children in CDA treatment group and 17 deficient children in other treatment groups had a 22% power to detect a difference of 26% using the two-sided Fisher’s exact test. With such sample sizes, the difference should be at least 57% to reach a power of 80%. It should be noticed that only 7% of children were G6PD deficient (less than reported in [25,26]); the size of the effect (5/9-5/17) remains high even if not statistically significant.

The available information indicates that CDA in G6PD-deficient patients is associated with a substantial risk of haematological toxicity, due to an oxidant metabolite of dapsone causing haemolytic damages in these patients. Assuming the CDA development would have continued, it would have implied that, before any treatment, patients would have to be tested for G6PD deficiency, e.g. with the cheap and fast Beutler’s fluorescent test [27] judged nearly as good as the biomolecular (PCR/RFLP) method [28], a difficult measure to implement in busy outpatient clinics. In addition, a recent stability study observed a degradant in CDA which has the potential to limit the shelf-life of the product [29], rendering the deployment of this treatment even more difficult. When considering also the potential for drug resistance, CDA may not successfully treat patients carrying parasites with the *Pfdhfr* I164L mutation, mainly found in Asia while this particular mutation seems rare in sub-Saharan Africa [30,31]. For the aforementioned reasons, stopping the development of CDA appears as the only possible decision.

## Competing interests

UDA has received travel grants from sanofi Aventis, sigma tau and Novartis. He has also received research funds from sanofi Aventis and sigma tau.

## Authors’ contributions

UDA and JPVG designed the sub-study. QB/SM/RG, AT/AY and CN/PP/AD/ET were responsible for the study implementation and data collection in Manhiça, Jinja/Tororo and Mbarara, respectively. PF, CVO and CVM conceived and performed the G6PD experiments. HvL managed the data. JPVG and CVM analysed the data. ARR supervised data analyses and results reporting. CVM, JPVG and UDA wrote the paper and all authors reviewed the manuscript.
